# Harnessing the power of new genetic tools to illuminate *Giardia* biology and pathogenesis

**DOI:** 10.1093/genetics/iyae038

**Published:** 2024-04-16

**Authors:** Kari D Hagen, Christopher J S Hart, Shane G McInally, Scott C Dawson

**Affiliations:** Department of Microbiology and Molecular Genetics, University of California, Davis, Davis, CA 95616, USA; Department of Microbiology and Molecular Genetics, University of California, Davis, Davis, CA 95616, USA; Department of Biology and Biotechnology, Worcester Polytechnic Institute, Worcester, MA 01609, USA; Department of Microbiology and Molecular Genetics, University of California, Davis, Davis, CA 95616, USA

**Keywords:** *Giardia*, CRISPR/Cas9, morpholino, CRISPRi, Cre-Lox

## Abstract

*Giardia* is a prevalent single-celled microaerophilic intestinal parasite causing diarrheal disease and significantly impacting global health. Double diploid (essentially tetraploid) *Giardia* trophozoites have presented a formidable challenge to the development of molecular genetic tools to interrogate gene function. High sequence divergence and the high percentage of hypothetical proteins lacking homology to proteins in other eukaryotes have limited our understanding of *Giardia* protein function, slowing drug target validation and development. For more than 25 years, *Giardia* A and B assemblages have been readily amenable to transfection with plasmids or linear DNA templates. Here, we highlight the utility and power of genetic approaches developed to assess protein function in *Giardia*, with particular emphasis on the more recent clustered regularly interspaced palindromic repeats/Cas9-based methods for knockdowns and knockouts. Robust and reliable molecular genetic approaches are fundamental toward the interrogation of *Giardia* protein function and evaluation of druggable targets. New genetic approaches tailored for the double diploid *Giardia* are imperative for understanding *Giardia*'s unique biology and pathogenesis.

## Introduction

The extraordinary diversity of eukaryotic life spans ∼8 primary lineages or “supergroups,” with the majority of supergroups represented solely by microbial eukaryotes (Burki 2014). Yet, much of this known eukaryotic diversity is not represented in cell biology research, which has predominantly focused on a select few “model” eukaryotic organisms that are primarily macroscopic animals, fungi, or plants. While the study of such models has been pivotal in advancing biomedical research, exploration of the genetics and cell biology of diverse cellular lifeforms, including both free-living and parasitic eukaryotic microbes, offers promise toward understanding novel cellular adaptations and evolution (Russell et al. 2017).

Knowledge of genetic and functional diversity across the tree of life can aid us in determining the true extent to which the biology of model organisms is representative of biological diversity. Many cellular phenomena that define microbial eukaryotes may be limited or absent in common model organisms, underscoring the need to develop genetic tools for “non-model” eukaryotes. For instance, the diplomonads, a subgroup of the eukaryotic supergroup termed Excavates, exhibit distinctive cellular traits such as the presence of 2 equivalent nuclei and 8 flagella. Known diplomonads are also anaerobes, having lost mitochondria while retaining mitochondria-related organelles in both free-living and parasitic species (Stairs et al. 2015). The development of molecular genetic tools has been limited in this diverse supergroup of microbial eukaryotes (Russell et al. 2017), with the exception of the diplomonad *Giardia lamblia*, a widespread intestinal parasite that causes diarrheal disease in humans and animals. With a global impact, *Giardia* infects over 300 million people annually, particularly in developing or developed countries with poor sanitation and inadequate water treatment (Hotez 2008). *Giardia* is unique in possessing an elaborate cuplike microtubule organelle, the ventral disc, which is required for the attachment to the host epithelium (Nosala et al. 2018). The specific molecular and cellular mechanisms underlying *Giardia's* pathogenesis in the host, including parasite colonization and differentiation into cysts in the gastrointestinal tract, remain unclear; thus, genetic tools and methodologies are essential to study basic *Giardia* biology.

Symptoms of giardiasis frequently include acute or chronic diarrhea, abdominal pain, bloating, nausea, and malabsorption, although *Giardia* infection may also be asymptomatic (Einarsson et al. 2016). In children, the consequences of chronic giardiasis can be severe and may include malnutrition, stunting, and cognitive delays (Berkman et al. 2002). *Giardia* infection can also have long-term impacts such as lactose intolerance (Hanevik et al. 2009; Halliez and Buret 2013), irritable bowel syndrome (IBS), and chronic fatigue (Hanevik et al. 2014; Hanevik et al. 2017). Infection begins with the ingestion of environmentally resistant *Giardia* cysts by the host. Cysts then transform into motile trophozoites as they transit into the gastrointestinal tract (Einarsson et al. 2016). The excysted trophozoites attach extracellularly to the gut epithelium via the ventral disc, primarily proliferating in and colonizing the small intestine. Unknown cues cause trophozoites to reencyst, and the cysts are disseminated in feces to infect new hosts (Einarsson et al. 2016). There is a growing need for new therapeutic interventions for giardiasis, as standard drugs such as metronidazole have failure rates of up to 40% (Nabarro et al. 2015), with growing evidence of drug resistance (Upcroft and Upcroft 2001). Candidate drug screening approaches have been extensively utilized to identify compounds targeting trophozoite viability, but the mechanisms of action for most anti-*Giardia* drugs remain poorly understood, with only oxidative stress (in the case of the nitroimidazoles) being strongly implicated (Emery et al. 2018; Riches et al. 2020; Krakovka et al. 2022). Despite recent high-throughput candidate screens (Tiash et al. 2020), the identification of novel druggable targets has lagged due to the prior lack of reliable approaches to disrupt gene expression in *Giardia*. Nevertheless, recent and ongoing efforts to develop more robust and reproducible genetic manipulation techniques in *Giardia* are significantly advancing our capability to investigate the basic biology and pathogenic mechanisms of this important diplomonad protist and intestinal parasite.

### Unique challenges to developing molecular genetic tools for double diploid *Giardia*

Polyploidy is common in many plants, fungi, and some metazoans including amphibians, and even in the era of clustered regularly interspaced palindromic repeats (CRISPR)/Cas9 genetic tools, the presence of multiple alleles for each genetic locus presents a significant challenge for genetic manipulation (Comai 2005). Polyploidy can offer selective advantages for organisms in that it can increase genetic diversity, and thus, polyploids can exhibit hybrid vigor or heterosis. Deleterious mutations in polyploids can also be masked by functional copies of the gene from other chromosome sets, reducing their phenotypic impact (Comai 2005).

Like other polyploids, the “double diploid” nature of diplomonads is problematic for developing genetic tools. *Giardi*a's 2 diploid nuclei are essentially genetically equivalent (Morrison et al. 2007; Hanevik et al. 2015; Klotz et al. 2023), and both are transcriptionally active (Kabnick and Peattie 1990). Aneuploidy has been reported, however (Tumova et al. 2016). Zoonotic *Giardia* is classified into 8 assemblages (A–H; akin to species) that include the assemblages A1 and A2 [e.g. strain WB (A1) and DH (A2)] and assemblages B1 and B2 (e.g. strains GS and H3) (Heyworth 2016) that are known to infect humans and other mammalian hosts. *Giardia* isolates from other assemblages (C–H) generally infect only other mammalian hosts. Assemblage B strains are generally characterized by extensive allelic heterozygosity (Franzen et al. 2009; Adam et al. 2013), whereas in contrast, assemblage A isolates tend to exhibit very little allelic heterozygosity. One notable exception is the recent identification of low allelic heterozygosity in a human B isolate (Klotz et al. 2023). Genome sequences are available for strain WB (Morrison et al. 2007; Xu et al. 2020), strain GS (Franzen et al. 2009; Adam et al. 2013), assemblage E strain P15 (Jerlstrom-Hultqvist et al. 2010), and additional *Giardia* clinical isolates (assemblages A and B) from humans (Hanevik et al. 2015; Klotz et al. 2023). Reasonably robust molecular genetic tools have only been developed for *Giardia* strains WB and GS, however (Singer et al. 1998). Cysts contain 4 nuclei due to an incomplete division prior to the assembly of the cyst wall (Einarsson et al. 2016), and the nuclei can fuse during encystation, which may homogenize the genetic content of daughter nuclei through recombination, a process termed “diplomixis” (Poxleitner et al. 2008; Carpenter et al. 2012).

The *G. lamblia* strain WB clone C6 (ATCC_50803) genome contains roughly 5,000 genes (Morrison et al. 2007), with over 3,500 expressed in trophozoites and 2,500 expressed in cysts (Rojas-López et al. 2021). In vitro encystation protocols have been developed for the WBC6 and GS isolates, yet these protocols have not been established for other isolates or assemblages. All assemblages appear to lack a nonhomologous end-joining (NHEJ) pathway and are proposed to lack a defined sexual cycle (Carpenter et al. 2012). Moreover, our understanding of *Giardia* transcription factors and promoters is still in its infancy, and the limited number of selectable markers has hindered our ability to experimentally modulate *Giardia* gene expression with high efficiency. Given these constraints and the potential genetic variations between assemblages, there is a pressing need to develop molecular genetic tools for representative isolates from all *Giardia* assemblages that infect humans.

Omics-based approaches for genome functional annotation are heavily reliant upon comparative inferences of protein homology, and thus, it is often challenging to predict protein function in non-model organisms or eukaryotes like *Giardia* that are from evolutionarily distant groups. Genomes from multiple *Giardia* assemblages have been sequenced, yet most protein functions are predicted based on comparative annotations rather than definitive experimental determinations of function. This difficulty is compounded by the extreme divergence of *Giardia* proteins from known homologs in well-studied eukaryotes and the significant portion (over 42%) of expressed genes in *Giardia* that are classified as “hypothetical,” lacking homology to any known protein in other organisms (Xu et al. 2020). Furthermore, many abundant *Giardia* proteins lack identifiable conserved protein motifs, and there are several sizeable gene families (e.g. annexins, NEK kinases, and ankyrin repeat proteins) whose functional predictions are challenging based on homology alone (Xu et al. 2020). Despite the difficulty of in silico functional predictions, the extreme sequence divergence—even in genes with human homologs—offers unique possibilities for novel druggable targets. In any event, functional analyses of *Giardia* proteins essential to the parasite life cycle are fundamental toward understanding pathogenesis and developing new classes of anti-*Giardia* compounds (Riches et al. 2020).

### Tagging approaches to ascertain protein function in live and fixed *Giardia*

**Fig. 1.**
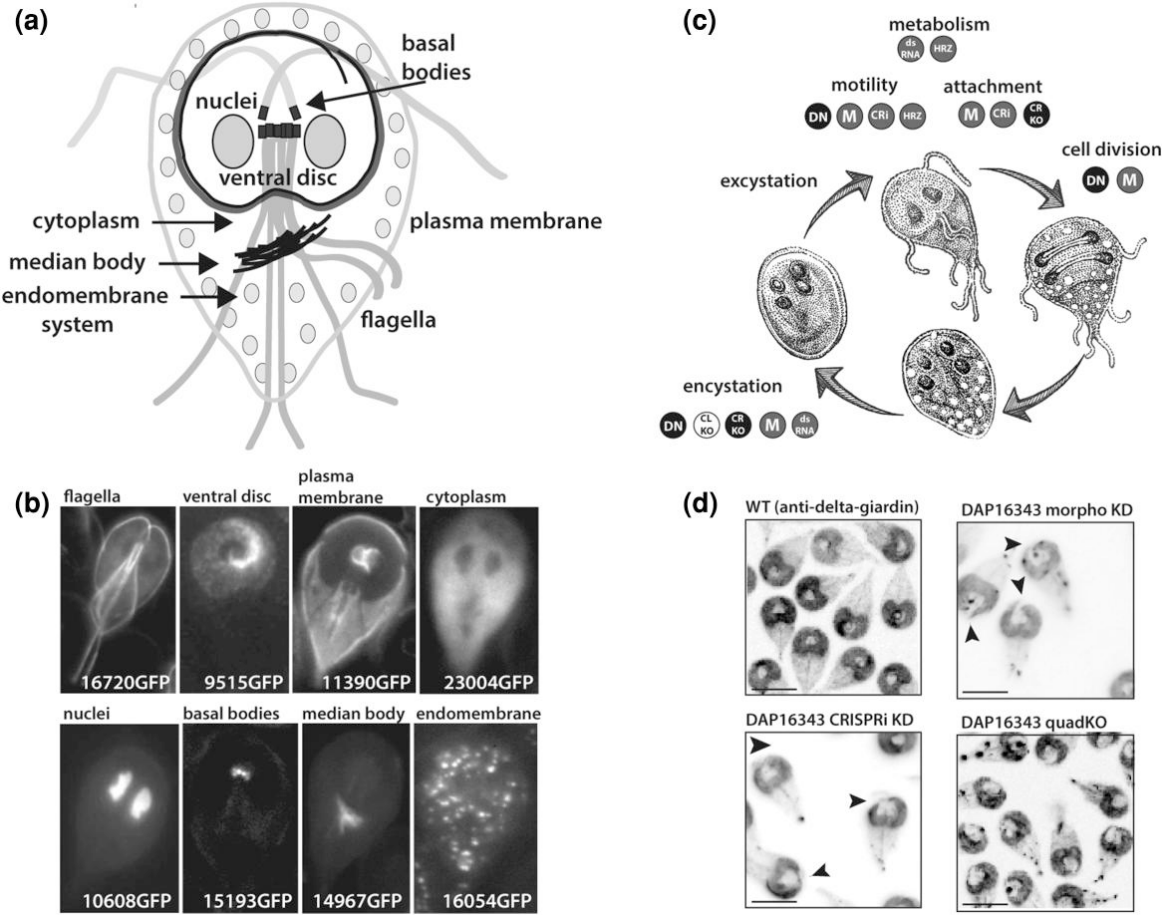
Molecular genetic tagging and gene depletion methodologies throughout the *Giardia* life cycle. In a) and b), C-terminal GFP-tagged hypothetical proteins (16720GFP, 9515GFP, 23004GFP, 10608GFP, 15193GFP, 14967GFP, and 16054GFP) and a member of the understudied yet abundant NEK kinase protein family (11390GFP) localize to distinct subcellular structures of the trophozoite, facilitating the understanding of their functions in *Giardia*. In c), the *Giardia lamblia* life cycle is shown, along with the molecular genetic approaches that have been used to understand various cellular processes in the cycle. Knockdown strategies include transcriptional knockdown methods such as hammerhead ribozyme (HRZ), the overexpression of dsRNA (dsRNA), and CRISPR interference (CRi), as well as translational knockdown with morpholinos (M) and the overexpression of dominant negative (DN) proteins. Gene knockout approaches include Cre-Lox-mediated marker recycling (CL KO) and CRISPR/Cas9-mediated HDR (CR KO). In d), representative images show transcriptional (CRISPRi) and translational (morpholino) knockdown and CRISPR/Cas9-medidated knockout of 4 alleles (quadKO) of the disc-associated median body protein (DAP16343) compared with a wild-type disc structure. In each image, the disc is stained using anti-delta-giardin antibody against disc microribbons. Similar ventral disc defects are seen with CRISPRi or morpholino-based knockdowns (arrows), compared with the 100% penetrance of aberrant discs seen in CRISPR/Cas9 quadruple allelic knockouts. Scale bar equals 10 µm.

Determining the subcellular localization of a protein is often a first step in the cell biological analysis of protein structure and function, and epitope and fluorescent tagging approaches have been used extensively in live and fixed *G. lamblia* trophozoites and cysts since the initial demonstration of these methods by Elmendorf *et al*. (Elmendorf et al. 2000). The amitochondriate trophozoite has a uniquely complex subcellular structure that promotes the visualization and classification of tagged proteins to specific functional locations, including the plasma membrane, cytoplasm, ventral disc, 8 flagella, median body, endomembrane system, and the 2 nuclei (Fig. 1). While *Giardia* lacks canonical mitochondria, it is proposed to possess mitochondrion-derived organelles—mitosomes—whose cellular function is understudied; these unique organelles have also been characterized by protein localization of homologous mitochondrial proteins in *Giardia* (Martincová et al. 2012).

Genes encoding tagged proteins are transfected into *Giardia* trophozoites by electroporation. Robust transfection methods for the A1 and B assemblages have been available for over 25 years (Yee and Nash 1995; Singer et al. 1998), resulting in stable transfectants within several weeks under positive antibiotic selection. Assemblage A1 strains (e.g. WBC6) can maintain episomal plasmids or integrate linear dsDNA into the genome. In contrast, episomal plasmids are not known be maintained in assemblage B strains, but linear or circular dsDNA can be integrated into the genome of assemblage B strain GS (Yee and Nash 1995; Singer et al. 1998). Two selectable markers, *pac* (puromycin N-acetyltransferase) and *neo* (neomycin phosphotransferase), are widely used in *G. lamblia* strain WBC6 (A1 assemblage) (Singer et al. 1998; Davis-Hayman and Nash 2002). Transfected cells are selected using puromycin or neomycin (G418), respectively, and stable transfected strains can be maintained in culture under selection for weeks to months or may be stored indefinitely at −80°C or in liquid nitrogen (Davids and Gillin 2011). Stable episomal plasmids localize to only 1 of the 2 nuclei after transfection, and daughter cells inherit just 1 tagged nucleus (Sagolla et al. 2006). Both the maintenance of episomal plasmids and the integration of single copies of tagged genes into the genome by homologous recombination (Gourguechon and Cande 2011) have permitted protein tagging for both fluorescent immunostaining and electron microscopy of aldehyde or methanol-fixed samples. Gene expression can be controlled using *Giardia* promoters that differ in strength (Davis-Hayman and Nash 2002) or with a tetracycline-inducible expression system (Sun and Tai 2000). Lastly, beyond their use in localizing proteins, epitope tags such as tandem affinity purification (TAP) tags or streptavidin tags have been used successfully to identify and purify proteins and complexes in *Giardia* (Jerlström-Hultqvist et al. 2012).

Natively fluorescent protein tags such as eGFP, mNeonGreen, or mCherry have frequently been used to determine the localization of proteins in both live and fixed *Giardia* trophozoites or cysts (Dawson and House 2010). Despite their requirement for nanomolar concentrations of oxygen for proper folding, fluorescent tags do not require that *Giardia* be incubated extensively under highly oxic conditions. For example, maximal eGFP folding requires between 1 and 20 µm O_2_ or about 0.06 to 2% O_2_ at 37°C (Morin-Adeline and Slapeta 2016). This low concentration of oxygen is within the physiological range of the mammalian gut, where *Giardia* colonizes and thrives under slightly oxic conditions (from 0 to 45 µm O_2_ or between 0 and 4.4% O_2_) (Paget et al. 1993), and it is also near the range measured for low-oxygen *Giardia* growth medium (∼75–100 µm O_2_).

When imaging live parasites, physiological buffers must be devoid of high concentrations of autofluorescent protein components, and reducing conditions are required to reproduce physiological conditions as closely as possible (Dawson and House 2010). Fusion protein expression should be modulated using native promoters and/or the integration of single copies of tagged genes into the genome to prevent artifacts caused by protein aggregation or misfolding. Live *Giardia* can be imaged over time scales ranging from minutes to several hours, allowing the quantitative visualization of fast dynamic processes during flagellar assembly (McInally et al. 2019b) or of longer processes such as mitosis and cytokinesis (Hardin et al. 2017). Fluorescent tagging not only allows the localization of proteins in live cells but also permits the quantification of dynamic and stable protein pools using techniques such as fluorescence recovery after photobleaching (FRAP), as our lab has used for structural components of the ventral disc (House et al. 2011) or dynamic components of the 8 flagella (McInally et al. 2019b). Others have used this technique to study the dynamics of endocytosis (Gaechter et al. 2008). Fluorescent tags also allow the dynamic visualization of *Giardia* attachment (House et al. 2011), a key step in pathogenesis and interaction with the host.

The power of localization as a first step in ascertaining gene function in an understudied microbe was exemplified by our high-throughput cloning and imaging of the subcellular localization of over 600 C-terminal GFP tag fusions for the *Giardia* informatics resource (GiardiaDB.org). In this project, subcellular localization data for known and novel proteins was provided as it was generated, giving others insight into the functions of many *Giardia* proteins prior to peer-reviewed publication of this data (Warrenfeltz et al. 2018). We tagged primarily hypothetical or ankyrin repeat proteins and expressed the fusions from the native *Giardia* promoter regions (roughly 100–200 bp upstream of the transcription start site). Representative high-resolution images of the subcellular localizations of the GFP-tagged proteins have been deposited into GiardiaDB as a resource for the *Giardia* research community (see Fig. 1), along with protein localizations linked to the *Giardia* GO annotations. Plasmids for GFP-tagged *Giardia* strains are available from our lab upon request. For live imaging of subcellular localizations using fluorescent tags, trophozoites are imaged at 37°C in imaging medium in plates with coverglass bottoms or are embedded in 1% low-melt agarose to limit motility (McInally et al. 2019b). While GFP and similar fluorescent tags are larger than epitope tags, the larger size has rarely affected the localization of known *Giardia* proteins. In *Giardia*, mislocalized GFP fusions tend to be cytoplasmic and are often found in large cytoplasmic inclusions, rather than localized to specific cytoskeletal structures (Warrenfeltz et al. 2018).

In addition to fluorescent tags like GFP and mNeonGreen, other protein fusion tags such as SNAP-tag (New England Biolabs) or HaloTag (Promega) are also useful for imaging protein localization in both live and fixed *Giardia* (Regoes and Hehl 2005). These tags can be covalently attached to a variety of substrates, including diverse fluorophores with various emission spectra. One advantage of SNAP-tagged strains is the ability to anaerobically image *Giardia* in TYI-S-33 medium (Regoes and Hehl 2005). The proximity labeling tag APEX2 has also been optimized recently for *Giardia* and is particularly useful as a reporter for transmission electron microscopy (TEM) (Ástvaldsson et al. 2019). Finally, bioluminescent “bioreporter” tags such as firefly luciferase or NanoLuc can be fused to promoters or proteins to report transcriptional activity or protein expression levels in vitro or in vivo (Weissleder and Ntziachristos 2003). Bioreporters have been used successfully for in vitro expression monitoring in *Giardia* (Yee and Nash 1995), and we have recently developed these methods for in vivo and ex vivo imaging of *Giardia* infections (Barash et al. 2017). In vivo bioluminescent imaging (BLI) has been used in many parasites and pathogens for real-time and longitudinal monitoring of infection dynamics (Saeij et al. 2005). For in vivo BLI in *Giardia*, integrated luciferase bioreporter tags for metabolism and cyst development are used to track in vivo temporal infection dynamics in mice and gerbil animal models (Barash et al. 2017). Because in vivo studies of giardiasis are limited by the inaccessibility of the intestinal tract, these direct live imaging methods to quantify parasite infection dynamics augment standard indirect approaches to quantify in vivo parasite burden, differentiation, and physiology in euthanized study animals. Moreover, the use of ex vivo BLI in *Giardia*-infected animal models has permitted the quantitative visualization of spatial dynamics of *Giardia* colonization throughout the gastrointestinal tract, enabling precise anatomic sampling of in vivo *Giardia* gene expression (Pham et al. 2017).

### Transcriptional and translational “knockdown” approaches to assess protein function

RNA interference (RNAi) is a powerful tool for gene silencing in many parasites, yet RNAi is not efficient in *Giardia*, despite the presence of conserved components of RNAi machinery (Macrae et al. 2006). Alternative transcriptional repression methods such as the overexpression of long double-stranded RNAs (Rivero et al. 2010) or the Giardiavirus-mediated expression of hammerhead ribozymes (HRZs) have also been used for transcriptional knockdowns (Dan et al. 2000), yet neither has had widespread use or reproducibility.

Both morpholino-based knockdowns and more recent CRISPR interference (CRISPRi)-based knockdowns have been used extensively to evaluate the functional roles of cytoskeletal, cell cycle, and endomembrane proteins and transcription factors, as well as to define the roles of hypothetical proteins of unknown function in *Giardia* (Fig. 1 and Table 1). Obviously, not all knockdowns (or even gene knockouts) are expected to result in quantifiable phenotypes, and the identification of aberrant phenotypes will depend on the continued development of new phenotypic and quantitative assays in *Giardia*.

**Table 1.** Selected publications are categorized, highlighting either the development of a molecular genetic methodology (method development) or the use of a molecular genetic method to study processes in the *Giardia* life cycle, including metabolism, cell division (mitosis, cell cycle, and cytokinesis), attachment, motility, and functions of the endomembrane system and encystation.

	Method development	Metabolism	Cell division	Motility	Attachment	Endomembrane/encystation
Epitope tagging	Sun *et al*. 2003 PMID: 12686559 Gourguechon and Cande 2011 PMID: 21115739 Jerlström-Hultquist et al. 2012 PMID: 22611020 Zumthor *et al*. 2016 PMID: 27438602 Martincova *et al*. 2015 PMID: 26055323		Davids *et al*. 2008 PMID: 17964578 Smith *et al*. 2012 PMID: 22429767 Carpenter et al. 2012 PMID: 22366460 Vicente *et al*. 2014 PMID: 25057014 Kim *et al*. 2016 PMID: 27859890 Hardin et al. 2017 PMID: 28679631	Ebneter *et al*. 2016 PMID: 27976675	Palm *et al*. 2005 PMID: 15850703 Lauwaet *et al*. 2011 PMID: 21723868	Sun *et al*. 2002 PMID: 12421304 Gaechter et al. 2008 PMID: 17892527 Stefanic *et al*. 2009 PMID: 19622633 Lauwaet *et al*. 2011 PMID: 21723868 Faso *et al*. 2012 PMID: 23094658 Paredez *et al*. 2014 PMID: 24728194 Kim *et al*. 2016 PMID: 27859890 Zumthor *et al*. 2016 PMID: 27438602 Rout *et al*. 2016 PMID: 27926928 Hennessey *et al*. 2016 PMID: 27806042 Krtkova *et al*. 2016
						PMID: 27555307 Voleman *et al*. 2017
						PMID: 28372543 Cernikova *et al*. 2020 PMID: 32092130
Protein-fusion tags	Yu *et al*. 1995 PMID: 7651405 Yee and Nash. 1995 PMID: 7777558 Singer et al. 1998 PMID: 9574910 Yee et al. 2000 PMID: 10753960 Elmendorf et al. 2000 PMID: 10699262 Regoes and Hehl 2005 PMID: 16382896 Martincova et al. 2012 PMID: 22558433 Barash et al. 2017 PMID: 28656177 Spycher *et al.* 2013 PMID: 23617761		Sagolla et al. 2006 PMID: 17105767 Poxleitner et al. 2008 PMID: 18339940 Gourguechon *et al.* 2013 PMID: 23525017 House et al. 2011 PMID: 21829364 Dawson *et al.* 2007 PMID: 17766466 Kim *et al.* 2020 PMID: 33412772 Hardin et al. 2017 PMID: 28679631	Dawson *et al.* 2007 PMID: 17766466 Hoeng *et al.* 2008 PMID: 18463165 House et al. 2011 PMID: 21829364	Hagen *et al.* 2011 PMID: 22206034 Woessner *et al.* 2012 PMID: 22247266 Nosala et al. 2020 PMID: 32661087	Hehl *et al.* 2000 PMID: 10793152 Abodeely *et al.* 2009 PMID: 19749174 Martincova et al. 2012 PMID: 22558433 Faso *et al.* 2012 PMID: 23094658 Wampfler *et al.* 2014a PMID: 24747534 Wamplfer *et al.* 2014b PMID: 24732305 Zumthor *et al.* 2016 PMID: 27438602 Rout *et al.* 2016 PMID: 27926928 Krtkova *et al.* 2016 PMID: 27555307 Pham et al. 2017 PMID: 28620589 Barash et al. 2017 PMID: 28656177 Voleman *et al.* 2017 PMID: 28372543 Cernikova *et al.* 2020 PMID: 32092130 Shih *et al.* 2023 37945557
Inducible/overexpression	Sun and Tai 2000 PMID: 10613698	Leitsch *et al.* 2016 PMID: 27485086	Lauwaet *et al.* 2011 PMID: 21723868	Dawson *et al.* 2007 PMID: 17766466 Hoeng *et al.* 2008 PMID: 18463165 House et al. 2011 PMID: 21829364		Huang *et al.* 2008 PMID: 18768462 Krtkova *et al.* 2016 PMID: 27555307
dsRNA KD	Rivero et al. 2010 PMID: 20476805 Prucca *et al.* 2008 PMID: 19079052					Lauwaet *et al.* 2011 PMID: 21723868
Ribozyme KD	Dan et al. 2000 PMID: 10792730	Dan *et al.* 2000b PMID: 10924754 Feng *et al.* 2008 PMID: 18167307 Chen *et al.* 2007 PMID: 16982153 Saraiya *et al.* 2008 PMID: 19043559 Feng *et al.* 2010 21137307		Wei *et al.* 2010 PMID: 20515687		Castillo-Romero *et al.* 2010 PMID: 20532229
CRISPRi KD	McInally et al. 2019a PMID: 30379614 García-Huerta *et al*. PMID: 35952970			McInally et al. 2019a PMID: 30379614 McInally et al. 2019b PMID: 31855176	McInally et al. 2019a PMID: 30379614 Nosala et al. 2020 PMID: 32661087	Shih *et al.* 2023 37945557
Morpholino KD	Carpenter *et al.* 2009 PMID: 19377039		Vicente *et al.* 2014 PMID: 25057014 Kim *et al.* 2019 PMID: 30318753 Hardin et al. 2017 PMID: 28679631 Park *et al.* 2021 PMID: 33789729	Carpenter and Cande 2009 PMID: 19377039 House et al. 2011 PMID: 21829364 Kim *et al.* 2014 PMID: 24828878	Woessner *et al.* 2012 PMID: 22247266 McInally et al. 2019a PMID: 30379614 Kim *et al.* 2019 PMID: 30318753	Paredez *et al.* 2011 PMID: 21444821 Hennessey *et al.* 2016 PMID: 27806042 Krtkova *et al.* 2016 PMID: 27555307
Dominant negative	Dawson *et al.* 2007 PMID: 17766466 Gaechter et al. 2008 PMID: 17892527		House et al. 2011 PMID: 21829364 Kim *et al.* 2016 PMID: 27859890	Hoeng *et al.* 2008 PMID: 18463165 House et al. 2011 PMID: 21829364		Stefanic *et al.* 2009 PMID: 19622633 Voleman *et al.* 2017 PMID: 28372543 Gaechter et al. 2008 PMID: 17892527
Cre-Lox KO	Wamplfer *et al.* 2014a PMID: 24747534					Ebneter et al. 2016 PMID: 27976675
	Ebneter et al. 2016 PMID: 27976675					
CRISPR KO	Lin et al. 2019 PMID: 30856211 Horáčková et al. 2022 PMID: 35472287 Kim et al. 2022 PMID: 36207732 Hagen et al. 2023 DOI: 10.1101/2023.07.02.547441 Nosala et al. 2023 DOI: 10.1101/2023.07.04.547600				Hagen et al. 2023 DOI: 10.1101/2023.07.02.547441 Nosala et al. 2023 DOI: 10.1101/2023.07.04.547600	Lin et al. 2019 PMID: 30856211 Horáčková et al. 2022 PMID: 35472287 Kim et al. 2022 PMID: 36207732

### Translational knockdown using morpholinos

Although there are now effective methods for creating quadruple allele knockouts (nulls) in *Giardia* (see below), genetic approaches that go beyond simple gene knockout are crucial when studying the functions of essential genes, many of which could potentially disrupt parasite viability, attachment, or cell division. In *Giardia*, morpholino knockdown approaches have been available for over a decade (Carpenter and Cande 2009). Morpholinos are chemically modified antisense oligonucleotides that bind to mRNAs and sterically limit translation initiation complex formation, resulting in translational blockage without causing mRNA degradation. Moreover, their modified backbone is not recognized by nucleases (Krtkova and Paredez 2017).

This strategy for transient translational knockdown was developed by Carpenter and Cande for *Giardia* in 2009 and has been extensively used (Carpenter and Cande 2009). Given the short 5′ leader sequence in *Giardia* mRNAs, morpholinos are typically designed to target regions close to the transcriptional and translation start sites, often within the first few codons of a gene. Gene-targeted morpholinos are electroporated into trophozoites using standard methods. Knockdown phenotypes exhibit high penetrance (>60%) and can persist in the electroporated population for at least 48 h (Krtkova and Paredez 2017). As with other methods, both the specificity and potential for off-target binding of morpholinos should be evaluated with the use of mispaired morpholino controls. The downside to morpholino-based knockdown is that despite their effectiveness, morpholinos are diluted each generation. The transient effect of morpholino knockdown phenotypes makes this approach less suitable for studying infection dynamics of morpholino mutants in animal hosts. Additionally, the high cost of morpholino synthesis could be a limiting factor for genome-wide functional screens.

### Transcriptional knockdown using CRISPRi

The ability of Cas proteins to bind to target nucleic acid sequences and recruit a variety of effector proteins has been exploited not only for gene disruption but also for other molecular genetic and imaging methods. One such application is CRISPRi, which is a modification of the CRISPR/Cas9 system (Larson et al. 2013). CRISPRi utilizes a catalytically inactive, or “dead,” Cas9 protein (dCas9) to enable stable, inducible, or reversible transcriptional knockdown. CRISPRi has emerged as a robust alternative to RNAi for effectively silencing gene expression in both bacterial (Hawkins et al. 2015) and eukaryotic model systems (Larson et al. 2013), and in *Giardia* (McInally et al. 2019a), CRISPRi is a recently developed and powerful method for achieving stable and precise transcriptional knockdown in an effectively tetraploid protist. CRISPRi capitalizes on the gRNA’s ability to target inactive dCas proteins to specific genomic locations. Rather than catalyzing double-stranded breaks (DSBs), the inactive dCas9/gRNA complex prevents transcription initiation and/or elongation upon binding (Larson et al. 2013). In theory, all types of gene expression, including noncoding RNAs, microRNAs, antisense transcripts, and or polymerase III transcripts, could be targeted for repression. In model systems, CRISPRi is as effective as RNAi in transcriptional silencing, and fewer off-target effects have been reported (Larson et al. 2013).

In *Giardia*, CRISPRi directly and stably inhibits transcription and offers significant advantages over morpholino knockdown to repress both exogenous and endogenous genes (McInally et al. 2019a). Difficulties in targeting *Streptococcus pyogenes* Cas9 to the 2 nuclei were resolved by our lab by combining GFP nuclear localization data from tagged proteins with nuclear localization signal (NLS) prediction software to identify a *Giardia*-specific NLS that has since been added the C-terminal end of both Cas9 and the catalytically inactive dCas9 protein in addition to the commonly used SV40 NLS (McInally et al. 2019a). The CRISPRi episomal vector (dCas9g1pac) expresses dCas9 modified by the addition of this C-terminal *Giardia* NLS, as well as a specific gRNA targeting the gene to be knocked down, and the puromycin resistance gene, *pac*, for vector maintenance (McInally et al. 2019a). Complementary gRNA oligonucleotides are annealed and cloned into the CRISPRi vector using a 1-step digestion/ligation reaction. The modular design of the *Giardia* gRNA expression cassette allows for the concatenation of more than one gRNA to either target multiple sites in a single target gene, or target more than one *Giardia* gene or gene family (McInally et al. 2019a). Once imported into the nuclei, the dCas9/gRNA complex is targeted to a specific genomic locus, where it sterically interferes with RNA polymerase or transcription factor binding or with transcriptional elongation, as has been shown in bacteria or other eukaryotes (Larson et al. 2013).

To achieve maximal knockdown, several gRNAs should be tested for each target; the most efficacious gRNA should be used for further studies, as has been suggested for CRISPRi in human cells (Larson et al. 2013). gRNA design and cloning can be accomplished in a week, and CRISPRi strains are obtained about 2 weeks after electroporation of constructs into *Giardia* trophozoites, making this method scalable for limited screening of scores of genes or for large-scale random screening of phenotypes.

As with all genetic tools, it is important to underscore both limitations and potential caveats of CRISPRi or morpholino knockdown. In any organism, the phenotypic penetrance of CRISPRi or morpholino knockdowns is quantified as the proportion or frequency of mutant phenotypes observed in a population at the single cell level and complements the overall transcriptional knockdown quantified at the population level using methods such as quantitative RT-PCR. In *Giardia*, mutant phenotypic penetrance has been an issue in morpholino-based or CRISPRi-based knockdowns (McInally et al. 2019a) but is much less of an issue in isogenic quadruple knockouts (Hagen et al. 2023; Nosala et al. 2023). In a population, individual cells may have strong transcriptional knockdown with concomitantly stronger mutant phenotypes, whereas other cells have weaker transcriptional knockdown with weaker mutant phenotypes. Phenotypic penetrance of CRISPRi should thus be scored and correlated with dCas9 expression at the single cell level using anti-Cas9 antibodies. As an example, CRISPRi knockdown phenotypes for both a ventral disc protein and a flagellar assembly protein were observed to be more severe in cells with positive Cas9 antibody staining (McInally et al. 2019a).

At the population level, CRISPRi-based transcriptional knockdowns are nonetheless highly penetrant, and knockdown ranges from 10% to 90% depending on the choice of gRNA position (McInally et al. 2019a; Nosala et al. 2020). Rather than being only a deficit, these variations in CRISPRi knockdown levels highlight the overall tunability of this system and can be exploited for the evaluation of essential gene function. The ability to examine cells with varying degrees of knockdown and to reduce the severity of phenotypes by selecting gRNAs with less than complete transcriptional repression will be critical toward the evaluation of essential *Giardia* genes.

Another important consideration for CRISPRi is the loss of up to 90% of the transcriptional knockdown and mutant phenotype when strains are taken off positive selection due to the loss of the CRISPRi plasmid. Thus, while episomal CRISPRi-based knockdowns can be highly penetrant (McInally et al. 2019a), they are not necessarily stable in vivo for more than several days as CRISPRi strains lose the CRISPRi plasmids and thus mutant phenotypes are lost when removed from puromycin selection. For these reasons, the applicability of CRISPRi or morpholino knockdown mutants for in vivo animal or organoid infections of days to weeks is limited. Thus, knockout mutants, which are highly penetrant and stable off selection, are preferred for use in new in vivo infection models.

### New strategies for quadruple allele knockouts (nulls)

Double diploid, asexual trophozoites are effectively tetraploid, and a limited number of selectable markers combined with the need to knock out multiple gene copies have resulted in *Giardia* lagging far behind other protozoan parasites with respect to the use of molecular genetic approaches to define gene function. In theory, quadruple allelic knockouts could require up to 4 unique markers for positive antibiotic selection. To overcome this problem, Ebneter *et al*. (Ebneter et al. 2016) reported using a Cre/LoxP antibiotic marker recycling strategy to make the first quadruple allele knockout in *Giardia* trophozoites, disrupting CWP1, a component of the parasite cyst wall. While overcoming issues regarding antibiotic marker availability, this recycling strategy was time-consuming and laborious, so it has not been widely adopted.

CRISPR/Cas9 genome editing has revolutionized genetics in diploid and polyploid organisms (Barrangou and Doudna 2016). CRISPR knockout strategies have now been adapted for the double diploid *Giardia*, albeit with initial limited success. In most organisms, the Cas9/gRNA complex binds and induces a DSB that can be repaired in 2 ways—by NHEJ, which is error-prone and produces indels and frameshifts at the DSB that result in gene mutations or knockout, or by error-free homology-directed repair (HDR), which occurs at a low frequency if a gene-specific single- or double-stranded DNA homology repair template is supplied. HDR templates for gene knockout typically consist of a selectable marker flanked by homology arms matching the DNA sequence on either side of the DSB.

In the absence of the NHEJ pathway for DSB repair in *Giardia* (Morrison et al. 2007), indels and frameshift mutations causing gene knockout are not expected to occur at the endonuclease cleavage site. Lin *et al*. initially expressed Cas9 exogenously along with an HDR template containing the *pac* antibiotic resistance marker to knock out several copies of the myeloid leukemia factor (MLF)-like gene (GL50803_16424) in *Giardia* that is upregulated during antibiotic selection and encystation (Lin et al. 2019). Complete gene knockout was not achieved, likely due to the lack of a functional NLS localizing Cas9 to the nuclei, yet the integration of the *pac* cassette into at least 1 copy of *mlf* occurred, with a corresponding decrease in *mlf* transcripts and protein abundance.

More recently, using a modification of the CRISPRi plasmid that contains a *Giardia*-specific NLS to localize Cas9 to nuclei (McInally et al. 2019a), Horáčková *et al*. reported CRISPR/Cas9-based knockout by integrating a single linear antibiotic cassette into all 4 alleles with as little as 150 bp HDR template regions (Horáčková et al. 2022). This group confirmed the knockout of 2 interphase genes (*mem* and *mlf1)* and 1 encystation gene *(cwp1)* required to build cyst walls (Horáčková et al. 2022). Yet, for the putatively essential gene, *tom40*, the complete knockout of all 4 alleles with this single integrated marker strategy was not successful (Horáčková et al. 2022). Further, despite confirmation of knockouts using this single-marker strategy, no adverse or strong phenotypes were reported for any of the 4 genes.

Partial or complete knockouts of a gene with important cellular functions is likely to have a fitness cost, making the partial or complete mutant less competitive when grown as mixed population under selection. The use of additional selectable markers allows the selection of mutants that are less fit. Kim *et al*. used 2 antibiotic resistance markers (neomycin and blasticidin) to successfully knockout the encystation-specific gMyb2 (GL50803_8722) transcription factor, resulting in mutants that have reduced encystation efficiency (Kim et al. 2022). Building on our methods for stable CRISPRi transcriptional knockdowns in *Giardia*, our lab has recently developed new antibiotic markers to routinely create CRISPR/Cas9-mediated quadruple allele knockouts (nulls) within 6–8 weeks (Hagen et al. 2023). Using up to 3 positive selectable markers for the 4 alleles, we confirmed severe structural and functional phenotypes for 2 disc-associated proteins (DAPs) (Hagen et al. 2023, Nosala et al. 2023). Thus, the discrepancy between the single and multiple selectable marker approaches is likely due to the increased fitness costs of mutants with strong phenotypes for which additional selectable markers are required.

Currently, our multiple marker strategy requires at least 3 sequential electroporations—the first to create a strain expressing Cas9 and the gRNA and at least 2 more to introduce selectable markers for gene knockout followed by subsequent cloning of isogenic lines. Episomally expressed wild-type genes can be used to complement knockouts (Kim et al. 2022, Hagen et al. 2023, Nosala et al. 2023), and if needed, additional tagged cellular markers (e.g. mNeonGreen or luciferase) can either be integrated simultaneously during knockout with the selectable markers (Nosala et al. 2023) or introduced into knockouts via plasmids (Hagen et al. 2023). For example, to image aberrant disc movements and contractility, we knocked in an mNeonGreen-tagged disc edge marker (DAP12139mNG) in combination with knockout of the DAP7268 gene (Nosala et al. 2023). This approach would also be useful when applied to in vivo infection studies where the integration of luciferase markers (Barash et al. 2017) during knockout will facilitate the use of BLI strategies to compare differences in infection dynamics between mutant and wild-type *Giardia*.

## Conclusion

Molecular genetic tools that allow stable, precise, and robust knock-in, knockdown, and knockout of single or multiple genes are essential not only for understanding the pathogenesis of *Giardia* but also for transforming our knowledge of the molecular, cellular, and evolutionary biology of this unique microbial eukaryote. Because variations in the degree or timing of human and experimental infections have been associated with different *Giardia* assemblages, ongoing work should also aim to develop genetic tools in other *Giardia* strains, particularly B assemblages, that are of clinical relevance (Klotz et al. 2023). Thus, with CRISPR-mediated knockout mutants, we are finally able to test hypotheses of pathogenesis using innovative new organoid and animal models of *Giardia* infections. By quantifying the effects of knockout mutants in *Giardia* infections and studying their impact on pathogenesis, we can explore potential new targets for drug development, such as the DAPs, which are required for parasite attachment, colonization, and proliferation.
